# Family Outcomes Associated With Occupational Trauma Exposure in Frontline Workers: Protocol for a Scoping Review

**DOI:** 10.2196/93712

**Published:** 2026-09-16

**Authors:** Nicola Cogan, Martyna Ross, Rachel Miller, Thomas Johnson, Zunaira Iqbal, Alison Kirk, William Hodgson, Spence Whittaker, Andy Siddaway, Amalie Skovgaard, Patricia Graham

**Affiliations:** 1 NHS Lanarkshire Psychological Services, NHS Lanarkshire, Lanarkshire, Scotland, United Kingdom; 2 University of Strathclyde, 16 Richmond St, Glasgow, Scotland, G1 1XQ, United Kingdom, 44 141 552 4400

**Keywords:** family members, frontline workers, secondary traumatic stress, trauma, mental health, scoping review

## Abstract

**Background:**

Frontline workers are routinely exposed to traumatic and highly stressful events. Although the psychological effects of occupational trauma are well established, evidence suggests that these effects extend beyond the family members, affecting their psychological health, relationships, and functioning. Reported outcomes include secondary traumatic stress, psychological distress, caregiver burden, relationship difficulties, disrupted family functioning, and broader social impacts. However, evidence remains fragmented across occupational groups, disciplines, methodological approaches, and sociocultural contexts, limiting understanding of how occupational trauma affects families and hindering the development of family-inclusive, trauma-informed policies and services.

**Objective:**

This scoping review aims to systematically map and synthesize evidence on family outcomes associated with occupational trauma exposure among civilian frontline workers. It will characterize psychological, relational, social, and family functioning outcomes; examine variation across occupations, family roles, and sociocultural contexts; explore how family impacts have been conceptualized and measured; identify methodological approaches; and identify evidence gaps to inform future research, policy, and family-inclusive, trauma-informed practice.

**Methods:**

The review will be conducted according to the PRISMA-ScR (Preferred Reporting Items for Systematic Reviews and Meta-Analyses Extension for Scoping Reviews), the Joanna Briggs Institute methodological framework, and relevant elements of the PRISMA-S (Preferred Reporting Items for Systematic Reviews and Meta-Analyses Literature Search Extension). Comprehensive searches will be conducted from database inception to the final search date in PubMed (MEDLINE), PsycINFO, CINAHL, Web of Science, and Scopus. Eligible qualitative, quantitative, and mixed methods primary research will examine psychological, relational, social, or family functioning outcomes among family members of civilian frontline workers. Two reviewers will independently screen studies and chart data using a standardized framework, with disagreements resolved through discussion or consultation with a third reviewer. Evidence will be synthesized using narrative synthesis and evidence mapping.

**Results:**

Protocol development was undertaken between December 2025 and July 2026, including preliminary scoping searches; refinement of the Population, Concept, and Context (PCC) framework and eligibility criteria; development of the search strategy; stakeholder consultation; and piloting of the data-charting framework. No specific external funding was received for this review. As this is a scoping review, participant recruitment is not applicable. At the time of protocol submission, formal database analysis had not commenced. Comprehensive database searches and study screening are projected to commence in August 2026, with data charting and evidence synthesis undertaken between September and October 2026. Completion of the review is anticipated by November 2026, with publication of the review findings expected by the end of 2026.

**Conclusions:**

This review will comprehensively map how family outcomes associated with frontline occupational trauma have been conceptualized, measured, and reported across diverse civilian frontline occupations. It will identify evidence gaps, inform future research priorities, and support the development of family-inclusive, trauma-informed policies, services, and interventions for frontline workers and their families.

## Introduction

### Rationale for the Scoping Review

Frontline workers, including health care professionals, emergency service personnel, police officers, firefighters, and humanitarian responders, are routinely exposed to traumatic and highly stressful events as part of their occupational roles [1]. These exposures include repeated encounters with serious injury, death, violence, and human suffering, often under conditions of sustained workload pressure, moral complexity, and limited opportunity for recovery. The psychological consequences are well established and include posttraumatic stress disorder (PTSD), depression, burnout, moral injury, and increased risk of suicidality [2,3]. However, trauma rarely remains confined to the individual worker [4]. Growing evidence indicates that occupational stress and trauma exposure among frontline workers can have significant ripple effects across family systems, affecting the psychological well-being, relationships, and daily functioning of spouses, partners, and children [5,6]. Partners may experience secondary traumatic stress, emotional exhaustion, relationship strain, and increased caregiving responsibilities, whereas children may be affected through reduced parental availability, emotional withdrawal, disrupted parenting practices, and the intergenerational transmission of stress-related coping patterns [7-9].

Qualitative research has further illuminated these relational impacts. An interpretative phenomenological analysis of partners of veterans with mental health difficulties identified sustained caregiving, emotional containment, and advocacy as central features of partners’ experiences, illustrating how trauma shaped everyday relationships and family life [10]. Although conducted in a military context, these findings offer insights relevant to civilian frontline families. Civilian evidence has since strengthened this picture: a systematic review by Tekin et al [11] found that occupational stress among health care workers was associated with adverse psychological outcomes and poorer family functioning, including impaired communication, disrupted social life, and increased household burden, while a UK mixed methods study reported clinically significant levels of secondary traumatic stress among household members of health care workers, particularly spouses and partners [12]. Qualitative work has similarly shown that duty-related trauma becomes embedded in everyday family life, shaping communication, emotional availability, and shared understandings of “normal” family functioning [13]. Collectively, these findings confirm that the family-level impact of frontline occupational trauma is an increasingly important area of research and intervention development [14,15], extending beyond military populations into civilian health care and emergency service settings.

Despite this growing recognition, the evidence remains fragmented across disciplines, including occupational health, nursing, psychiatry, psychology, and family studies, and is characterized by substantial heterogeneity in study populations, frontline occupations, conceptual frameworks, methodologies, and outcomes. This fragmentation makes it difficult to establish which family outcomes are most consistently reported and where the evidence base remains thin, limiting the development of cumulative knowledge and the translation of evidence into family-inclusive policy, practice, and service design.

Existing reviews offer valuable but partial syntheses. Casas and Benuto [16] examined traumatic stress spillover among first responder families but were limited to 16 studies published through 2020, focused primarily on spouses of police, fire, and emergency medical personnel, and relied predominantly on cross-sectional evidence. May et al [4] synthesized qualitative evidence on family members of emergency first responders with diagnosed or probable PTSD, identifying themes of role change, secondary and vicarious trauma, child impacts, and unmet support needs, but were restricted to qualitative studies and a PTSD-specific sample. Neither review captures the broader, rapidly expanding evidence base across diverse civilian frontline occupations, trauma conceptualizations beyond PTSD, multiple methodological approaches, or postpandemic research, and no existing synthesis systematically examines how family impacts have been conceptualized and measured across sociocultural contexts.

A scoping review is therefore warranted to systematically map and synthesize the existing evidence on family outcomes associated with frontline workers’ occupational trauma. By integrating qualitative, quantitative, and mixed methods research across diverse civilian frontline occupations, this review will clarify the breadth and characteristics of the evidence base, examine how family impacts have been conceptualized and measured, identify knowledge gaps across populations and contexts, and inform future research, policy, and the development of family-inclusive, trauma-informed services and interventions [17].

### Scoping Review Questions

The primary question of the scoping review is as follows:

- What is known from the existing literature about family outcomes associated with occupational trauma exposure among civilian frontline workers?

The secondary questions of the scoping review are as follows:

- How do family outcomes associated with frontline workers’ occupational trauma vary across different frontline occupations, family roles, and sociocultural contexts?
- What psychological, relational, social, and family functioning outcomes have been examined among family members of frontline workers?
- How have family outcomes been conceptualized and measured across the existing literature?
- What methodological approaches have been used to investigate family outcomes associated with frontline workers’ occupational trauma?
- What gaps exist in the current evidence base, and what are the implications for future research, policy, and family-inclusive, trauma-informed practice?

## Methods

### Methodological Framework and Reporting Guidelines

This scoping review will be conducted in accordance with the PRISMA-ScR (Preferred Reporting Items for Systematic Reviews and Meta-Analyses Extension for Scoping Reviews) [18] and the Joanna Briggs Institute (JBI) methodological framework and will be informed by best-practice guidance for evidence synthesis [19]. Additionally, the conduct and reporting of the review will be guided by the evidence-based checklist for improving the quality of scoping reviews [20], which provides practical recommendations to enhance methodological transparency, consistency, and reporting standards. The PRISMA-ScR checklist is provided in Checklist 1.

### Ethical Considerations

This scoping review synthesizes evidence from previously published sources and does not involve primary data collection with human participants; therefore, formal ethical approval was not required.

### Consultation With Family Members and Stakeholders

Consistent with JBI guidance for scoping reviews, informal stakeholder consultations were undertaken between January and March 2026 to enhance the relevance, scope, and applicability of the review [21]. In total, 6 stakeholders participated in the consultation process, comprising 3 frontline workers from civilian frontline occupations and 3 family members of frontline workers with lived experience of the family impacts of occupational trauma. Participants were purposively identified through the authors’ professional networks and existing academic and clinical collaborations because of their relevant professional or lived experience.

The consultation process comprised 6 individual online discussions conducted via Microsoft Teams, each lasting approximately 30 to 60 minutes. Discussions focused on the relevance of the proposed review, the clarity and scope of the review questions, the appropriateness of the population, concept, and context (PCC) framework, terminology relating to frontline occupational trauma and family outcomes, and perceived gaps within the existing literature. Participants also highlighted family outcomes they considered particularly important, including psychological well-being, relationship functioning, family communication, caregiving burden, secondary traumatic stress, parenting, and broader family functioning. Feedback from these consultations informed the refinement of the review questions, eligibility criteria, the PCC framework, the preliminary search strategy, and the data-charting framework (Table 1). The consultations also contributed to refining the conceptualization of family outcomes and to ensuring that the protocol addressed issues considered meaningful and relevant to frontline workers and their families.

The consultations were undertaken solely to inform protocol development and did not form part of the research data included within the scoping review. No interviews or focus groups were conducted for research purposes, no audio recordings or transcripts were produced, no identifiable participant information was collected or retained, and no data were systematically analyzed or reported. Consequently, formal ethical approval was not required.

**Table 1.** Planned data charting framework.

Data category	Description
Authors, year	First author and year of publication
Country	Country or countries in which the study was conducted
Study aims	Study objectives, aims, or research questions
Study design	Overall study design (qualitative, quantitative, or mixed methods)
Methodology	Data collection methods and analytical approach
Family member population	Type of family members included (eg, spouse, partner, child, parent, sibling, and household member)
Participant characteristics	Demographic information, including age, relationship to the frontline worker, and sample size
Frontline occupation	Occupation of the frontline worker (eg, health care, emergency services, policing, firefighting, correctional services, and humanitarian work)
Trauma or occupational stress exposure	Type and nature of occupational trauma or stress exposure experienced by the frontline worker
Family outcomes	Psychological, relational, social, behavioral, physical, and family functioning outcomes reported
Theoretical or conceptual framework	Theoretical or conceptual framework underpinning the study, where reported
Key findings	Principal findings relating to family experiences and outcomes
Implications	Implications for research, policy, practice, or service development identified by the authors
Research gaps and limitations	Research gaps, methodological limitations, and future research priorities identified by the authors

### Eligibility Criteria

#### Population

The population of interest comprises family members of civilian frontline workers, defined broadly as spouses, partners, parents, siblings, children, and significant others living in the same household, with no age restriction. Frontline workers are defined as individuals in civilian roles involving routine, direct exposure to crisis, trauma, or emotionally demanding situations, including, but not limited to, health care professionals, emergency services personnel, humanitarian workers, correctional personnel, and social workers.

Studies will be included where family members are the primary focus, and outcomes relate explicitly to the psychological, relational, or social impacts of frontline occupational trauma. Studies focused on workers themselves or on students or trainees not undertaking direct frontline duties will be excluded. Occupational stress will be included only when explicitly linked to trauma exposure or cumulative occupational adversity rather than general workplace stress.

Families of military personnel will be excluded, as the military family literature constitutes a distinct, well-established evidence base with different organizational structures and support systems; this is a deliberate scoping decision to preserve conceptual coherence. Restricting inclusion to studies with a primary family focus may exclude findings reported as secondary outcomes in worker-focused research, a limitation partially mitigated through reference list screening of included studies and relevant reviews.

#### Concept

The concept explored in this scoping review is the impact of frontline worker trauma on family members. This includes a broad range of psychological, emotional, relational, and social outcomes associated with indirect exposure to occupational trauma and related secondary stress processes, such as secondary traumatic stress, chronic caregiving burden, emotional strain, and cumulative stress within family systems. Eligible studies will focus on how trauma experienced by frontline workers affects their partners, children, and other family members, including the ways in which trauma-related symptoms, stress responses, and coping strategies may be transmitted, shared, or internalized within the household. This may include changes in emotional availability, family functioning, communication patterns, and relational dynamics over time. Studies that concentrate primarily on the direct traumatic experiences or psychological outcomes of frontline workers themselves will be excluded, as this would shift the emphasis away from the review’s relational and family-focused perspective. Similarly, research centered on interventions designed exclusively to support frontline workers will not be included unless family member outcomes are explicitly reported and constitute a central focus of the study. The review will also exclude studies in which primary outcomes are purely biomedical or physiological, unless these outcomes are clearly linked to psychological, relational, or social impacts on family members.

#### Context

This scoping review will include studies conducted within both clinical and nonclinical frontline occupational contexts. These contexts encompass a broad range of roles in which individuals are routinely exposed to crisis, distress, or emotionally demanding situations, including, but not limited to, health care services, emergency response settings, mental health services, and other civilian frontline environments. Studies from all cultural and geographical settings will be eligible for inclusion in order to capture the global diversity of frontline work and family experiences. Including evidence from a wide range of sociocultural contexts will enable exploration of how cultural norms, family structures, and service systems may shape the ways in which frontline worker trauma is experienced and managed within families, as well as how impacts on family members may vary across settings.

#### Date of Publication

Electronic database searches will be conducted from database inception to the final search date, with no publication date restrictions applied. This decision reflects the gradual emergence of research on family-level impacts of frontline occupational trauma, which shifted over time from an initial focus on individual worker psychopathology toward more systemic and relational perspectives, including secondary traumatic stress, vicarious trauma, and caregiver burden. An open date range therefore ensures the review captures this full evolution of the evidence base while maintaining relevance to contemporary clinical practice and policy.

#### Types of Evidence Sources

This scoping review will focus exclusively on primary empirical research studies in order to map the research evidence base on the impacts of frontline worker trauma on family members. Policy documents, organizational reports, gray literature, commentaries, and conceptual or theoretical papers will not be included. Eligible studies will comprise qualitative, quantitative, and mixed methods primary research examining the experiences, well-being, or functioning of family members of frontline workers in relation to occupational trauma exposure. Qualitative studies may include interviews, focus groups, ethnographic research, or qualitative components of mixed methods studies exploring family members’ perceptions, experiences, and meaning-making. Quantitative studies will include observational designs such as cross-sectional surveys, cohort studies, and longitudinal studies reporting psychological, relational, or social outcomes among family members. Mixed methods studies integrating qualitative and quantitative approaches will also be included. There will be no restrictions on outcome measures, provided that the outcomes relate primarily to family members rather than frontline workers themselves. Experimental or interventional studies will be included only where outcomes specific to family members are reported. Review articles, commentaries, editorials, and opinion pieces will be excluded; however, reference lists of relevant reviews will be screened to identify additional eligible primary studies.

#### Language

Only studies published in English will be included. This restriction is unlikely to substantially bias the findings, as empirical evidence suggests that English-language restrictions in evidence syntheses do not systematically alter effect estimates or conclusions.

### Search Strategy

The search strategy will be developed and conducted in accordance with the JBI methodological guidance for scoping reviews and will be reported in accordance with the PRISMA-ScR and relevant elements of the Preferred Reporting Items for Systematic Reviews and Meta-Analyses Literature Search Extension (PRISMA-S) guidance to enhance transparency and reproducibility of the search process [18,22].

Protocol development commenced in December 2025 and included preliminary exploratory searches undertaken in CINAHL and PsycINFO to identify relevant keywords, index terms, and subject headings related to frontline worker populations, occupational trauma and stress exposure, and family members. The search strategy was subsequently refined through iterative discussion within the research team, consultation with an academic librarian with expertise in systematic and scoping review methodology, and stakeholder consultation undertaken between January and March 2026. These activities were undertaken to optimize the sensitivity, specificity, and comprehensiveness of the final search strategy. The final search strategy combines three core concepts:

1. Civilian frontline worker populations (eg, health care professionals, emergency service personnel, police officers, firefighters, paramedics, correctional personnel, humanitarian workers, and other civilian frontline occupations).
2. Occupational trauma and stress exposure (eg, trauma, traumatic stress, secondary traumatic stress, cumulative trauma, occupational adversity, compassion fatigue, moral injury, and posttraumatic stress).
3. Family members (eg, spouses, partners, children, parents, siblings, household members, caregivers, and significant others).

Comprehensive electronic database searches will be undertaken following publication of this protocol in PubMed (MEDLINE), PsycINFO, CINAHL, Web of Science, and Scopus. Searches will be conducted from database inception to the final search date. No publication date restrictions will be applied. Searches will be limited to studies published in English. Both controlled vocabulary terms (eg, MeSH and database-specific subject headings) and free-text keywords will be used where appropriate. Boolean operators (AND and OR), truncation, phrase searching, field-specific searching, and database-specific syntax will be applied to maximize search sensitivity and reproducibility.

To enhance transparency and reproducibility, the complete search strategy for PubMed (MEDLINE) is provided in Multimedia Appendix 1. The PubMed (MEDLINE) search strategy serves as the reference strategy for the review and will be adapted for PsycINFO, CINAHL, Web of Science Core Collection, and Scopus using each database’s controlled vocabulary, indexing terms, and search syntax while maintaining conceptual equivalence across the 3 core search concepts.

### Sources of Evidence

All retrieved records will be imported into reference management software, and duplicates will be removed. Title and abstract screening will be followed by full-text screening of potentially eligible studies. Screening will be undertaken independently by at least 2 reviewers, with discrepancies resolved through discussion or consultation with a third reviewer where necessary. Reference management will be conducted using EndNote (Clarivate), and screening will be managed using Rayyan. The study selection process will be documented using a PRISMA-ScR flow diagram.

### Data Extraction

Data will be extracted using a structured charting form developed specifically for this review and informed by the PCC framework and the review questions, consistent with established scoping review methodology [21,23,24]. The charting form will capture bibliographic information (author, year of publication, and country), study aims, study design and methodology, and participant characteristics, including family member type, age, and relationship to the frontline worker.

Data extraction will also include details relating to the frontline worker population, occupational setting, the type and nature of trauma or occupational stress exposure, and the conceptualization and measurement of family outcomes, including psychological, relational, social, behavioral, and family functioning outcomes. Information relating to theoretical or conceptual frameworks, key findings, implications for policy, practice, and service development, and identified research gaps and study limitations will also be extracted.

The data charting form will be piloted using a small sample of included studies to ensure clarity, consistency, and completeness before full data extraction commences [21,24]. Modifications to the charting form will be documented where necessary to improve data capture during the review process. Data extraction will be undertaken independently by 2 reviewers for a proportion of included studies to promote consistency, with disagreements resolved through discussion and, where required, consultation with a third reviewer. Extracted data will subsequently undergo descriptive numerical analysis, narrative synthesis, and evidence mapping, as described in the *Data Synthesis* section.

Consistent with the JBI methodological guidance for scoping reviews and the PRISMA-ScR, no formal critical appraisal of methodological quality or risk of bias will be undertaken [18,21]. The purpose of this scoping review is to systematically map the extent, characteristics, and nature of the existing evidence on the impacts of frontline workers’ occupational trauma on family members rather than evaluate the methodological quality or effectiveness of individual studies.

### Data Synthesis

Data synthesis will be undertaken in accordance with the JBI methodology for scoping reviews [21]. Findings will be synthesized using descriptive numerical analysis, narrative synthesis, and evidence mapping. Descriptive numerical analysis will summarize study characteristics, including publication year, country of origin, frontline occupation, study design, participant characteristics, family member type, trauma exposure, family outcomes, and methodological approach. Results will be presented in summary tables and descriptive figures. Narrative synthesis will integrate qualitative, quantitative, and mixed methods evidence to examine how family outcomes associated with frontline workers’ occupational trauma have been conceptualized, measured, and reported across occupational groups and sociocultural contexts. The synthesis will be structured around the review objectives, focusing on psychological, relational, social, behavioral, and family functioning outcomes. Evidence mapping will visually summarize the distribution of the literature across frontline occupations, family member groups, geographical settings, study designs, trauma-related concepts, and reported family outcomes. This will highlight areas where evidence is well established and identify important gaps. Evidence gaps relating to underrepresented populations, occupational groups, family member roles, geographical regions, methodological approaches, and outcome domains will be identified to inform future research, policy, practice, and the development of family-inclusive, trauma-informed services. Findings will be reported through narrative summaries, summary tables, and evidence maps in accordance with the JBI methodology [21] and PRISMA-ScR guidance [18].

### Deviations From Protocol

Any deviations from this protocol will be documented, justified, and reported in the final scoping review.

## Results

### Protocol Status and Preliminary Findings

Protocol development was undertaken between December 2025 and July 2026, including preliminary scoping searches, refinement of the PCC framework, development of the search strategy, stakeholder consultation, and piloting of the data-charting framework. Preliminary exploratory searches conducted in CINAHL and PsycINFO in December 2025 retrieved 557 records, of which 17 studies were identified as broadly relevant and used to refine the review’s scope, eligibility criteria, and search strategy; these preliminary findings do not constitute the results of the completed scoping review. No specific external funding was received for this review. As this is a scoping review, participant recruitment is not applicable. Comprehensive database searches, study screening, data charting, and evidence synthesis have not yet commenced and are projected to begin in August 2026, with data charting and evidence synthesis undertaken between September and October 2026. At the time of protocol submission, formal data analysis had not commenced. Completion of the review is anticipated by November 2026, with findings disseminated through peer-reviewed publication and conference presentations by the end of 2026.

Following publication of this protocol, eligible studies will then undergo title and abstract screening, full-text review, data charting, and narrative synthesis. The completed review will map the characteristics and distribution of the evidence base, identify knowledge gaps across occupations and sociocultural contexts, and inform recommendations for future research, policy, and the development of family-inclusive, trauma-informed interventions.

### Dissemination Plan

The dissemination strategy will be multifaceted, targeting academic, professional, policy, and public audiences with an interest in frontline well-being, occupational trauma, and family mental health, consistent with established knowledge translation frameworks [25,26]. Findings will be submitted to a peer-reviewed, open-access journal and reported in accordance with PRISMA-ScR [18], ensuring transparent reporting of the search strategy, study selection, data charting, and synthesis methods. Results will also be shared through poster and oral presentations at national and international conferences spanning psychology, occupational health, and trauma to reach researchers, clinicians, service leaders, and policymakers. Beyond academic channels, dissemination will include accessible evidence summaries and stakeholder briefings for frontline workers, family members, and frontline organizations to support practical interpretation of the findings. The review will also inform future research priorities and the development of family-inclusive, trauma-informed interventions, services, and policies [27].

## Discussion

### Principal Findings

This scoping review is expected to provide the first comprehensive map of evidence on family outcomes associated with occupational trauma exposure among civilian frontline workers. Based on preliminary scoping searches and the existing literature, family members are anticipated to report a broad range of adverse outcomes, including psychological effects such as secondary traumatic stress, anxiety, and depression; relational difficulties such as marital strain, disrupted communication, and family conflict; and broader social consequences, including increased household burden, role strain, and social withdrawal [28-30]. The review is also expected to show that the extent, consistency, and distribution of these outcomes vary considerably across frontline occupations, family roles, and sociocultural contexts and that this variation has not yet been systematically mapped. By identifying patterns within the existing evidence and highlighting areas in which evidence remains limited or absent, this review is expected to provide an important foundation for future research, policy development, and the advancement of family-inclusive, trauma-informed services and interventions. Two existing reviews have examined related questions but leave important gaps that this review is designed to address. Casas and Benuto [16] examined traumatic stress spillover among first responder families but were limited to 16 studies published through 2020 and focused primarily on spouses of police, fire, and emergency medical personnel using predominantly cross-sectional evidence. May et al [4] synthesized qualitative evidence on family members of emergency first responders with diagnosed or probable PTSD, identifying themes of role change, secondary and vicarious trauma, child impacts, and unmet support needs, but were restricted to qualitative studies and a PTSD-specific sample. More recent single-occupation studies, including the systematic review conducted by Tekin et al [11,12] and UK mixed methods survey of health care workers’ families, point to a rapidly expanding postpandemic evidence base that neither earlier review could capture. By integrating qualitative, quantitative, and mixed methods studies across diverse civilian frontline occupations, trauma conceptualizations beyond PTSD, and evidence published since the COVID-19 pandemic, the present review is positioned to substantially extend and update this earlier work rather than duplicate it.

### Strengths and Limitations

This review has several strengths. It will be conducted using a rigorous and transparent methodology consistent with the JBI framework for scoping reviews and reported in accordance with the PRISMA-ScR. The inclusion of qualitative, quantitative, and mixed methods studies will enable a more comprehensive synthesis than prior single-design reviews, and the use of evidence mapping alongside narrative synthesis will allow gaps to be identified systematically rather than descriptively.

Several limitations are also anticipated. The review will be restricted to English-language publications, which may exclude relevant non-English literature, although evidence suggests this is unlikely to systematically bias the conclusions. Consistent with scoping review methodology, no formal quality appraisal will be undertaken, meaning the review will map the extent and nature of the evidence rather than its methodological strength. Restricting inclusion to studies in which family members are the primary focus may exclude relevant findings reported as secondary outcomes within worker-focused research, a limitation partially mitigated through reference list screening. Families of military personnel will be excluded to preserve conceptual coherence with civilian frontline contexts, meaning findings may not generalize to military family populations. Finally, the stakeholder consultations informing this protocol were exploratory and involved a small, purposively identified sample; they were not intended as, and should not be interpreted as, formal qualitative research.

### Future Directions

The review will help identify priorities for future research, including the need for longitudinal studies, greater inclusion of children and other underrepresented family members, and improved consideration of sociocultural and contextual influences on family outcomes. From a policy and practice perspective, the findings will support the development of family-inclusive, trauma-informed approaches within frontline organizations by highlighting the importance of recognizing families as integral to the broader system affected by occupational trauma. Ultimately, this review is expected to strengthen the evidence base for understanding how frontline workers’ occupational trauma affects family members, while informing future research, guiding policy and service development, and supporting the implementation of family-inclusive, trauma-informed approaches across civilian frontline sectors.

## Supplementary material

10.2196/93712 Multimedia Appendix 1 Detailed search strategy.

10.2196/93712 Checklist 1 PRISMA-ScR checklist.

## Abbreviations

JBI: Joanna Briggs Institute
PCC: population, concept, and context
PRISMA-S: Preferred Reporting Items for Systematic Reviews and Meta-Analyses Literature Search Extension
PRISMA-ScR: Preferred Reporting Items for Systematic Reviews and Meta-Analyses Extension for Scoping Reviews
PSTD: posttraumatic stress disorder
