# Supplementary material for: Family Outcomes Associated With Occupational Trauma Exposure in Frontline Workers: Protocol for a Scoping Review
Source: JMIR Res Protoc. 2026 Sep 16;15:e93712. doi: 10.2196/93712 (PMC13581144; doi:10.2196/93712)
Supplement: Supplementary file 1 [file resprot-v15-e93712-s001.docx]

**Multimedia Appendix 1**

**Detailed Search Strategy**

*Family Outcomes Associated with Occupational Trauma Exposure in Frontline Workers: A Scoping Review Protocol*

**Search Strategy Development and Planned Electronic Database Searches**

Protocol development commenced in December 2025 and included preliminary exploratory searches conducted in CINAHL and PsycINFO to identify relevant keywords, index terms, subject headings, and database-specific terminology, and to assess the feasibility and scope of the evidence base. These searches retrieved 557 records (160 from CINAHL, 397 from PsycINFO). The 557 records were screened at title and abstract level, of which 17 studies were retained as broadly relevant to the review's scope. These preliminary results informed refinement of the review scope, PCC framework, eligibility criteria, and search strategy, and do not represent the findings of the completed review, which will follow the comprehensive search described below. The search strategy was subsequently refined through iterative discussion within the research team, consultation with an academic librarian with expertise in systematic and scoping review methodology, and stakeholder consultation undertaken between January and March 2026. Comprehensive database searches will be undertaken following publication of this protocol in the following databases: PubMed (MEDLINE), PsycINFO, CINAHL, Web of Science Core Collection, and Scopus.

Searches will be conducted from database inception to the final search date and will be limited to studies published in English. The search strategy combines three core concepts: (1) civilian frontline worker populations; (2) occupational trauma and trauma-related stress exposure; and (3) family members. Both controlled vocabulary terms (e.g., MeSH and database-specific subject headings) and free-text keywords will be used, as appropriate. Boolean operators (AND/OR), truncation, phrase searching, field-specific searching, and database-specific syntax will be applied to maximize search sensitivity and reproducibility.

**Search Documentation and Reproducibility**

Search strategy development commenced in December 2025 and progressed through iterative testing and refinement informed by preliminary exploratory searches, consultation with an academic librarian, and stakeholder consultation. No comprehensive database searches had been undertaken at the time of protocol development. Following publication of this protocol, comprehensive electronic database searches will be undertaken in accordance with the finalized search strategy. Retrieved records will be exported into EndNote for reference management, where duplicate records will be identified and removed before study selection. Title and abstract screening, followed by full-text screening of potentially eligible studies, will be undertaken independently by two reviewers using Rayyan. Disagreements will be resolved through discussion or, where necessary, consultation with a third reviewer. The study selection process will be documented using a PRISMA-ScR flow diagram.

**PubMed (MEDLINE) Search Strategy**

Database: PubMed (MEDLINE) | Platform: National Library of Medicine (PubMed) | Coverage: Database inception to the final search date | Language: English

| **Search No.** | **Search Strategy** |
| --- | --- |
| S1 | "Health Personnel"[Mesh] OR "Emergency Medical Technicians"[Mesh] OR "Firefighters"[Mesh] OR "Police"[Mesh] OR "Social Workers"[Mesh] OR "Rescue Work"[Mesh] |
| S2 | frontline worker*[Title/Abstract] OR front-line worker*[Title/Abstract] OR first responder*[Title/Abstract] OR emergency responder*[Title/Abstract] OR healthcare worker*[Title/Abstract] OR health care worker*[Title/Abstract] OR healthcare personnel[Title/Abstract] OR health care personnel[Title/Abstract] OR healthcare professional*[Title/Abstract] OR health care professional*[Title/Abstract] OR nurse*[Title/Abstract] OR physician*[Title/Abstract] OR doctor*[Title/Abstract] OR paramedic*[Title/Abstract] OR emergency medical technician*[Title/Abstract] OR EMT[Title/Abstract] OR firefighter*[Title/Abstract] OR police officer*[Title/Abstract] OR law enforcement[Title/Abstract] OR correctional officer*[Title/Abstract] OR prison officer*[Title/Abstract] OR social worker*[Title/Abstract] OR mental health worker*[Title/Abstract] OR humanitarian worker*[Title/Abstract] |
| S3 | S1 OR S2 |
| S4 | "Stress Disorders, Post-Traumatic"[Mesh] |
| S5 | trauma*[Title/Abstract] OR traumatic stress[Title/Abstract] OR occupational trauma[Title/Abstract] OR work-related trauma[Title/Abstract] OR duty-related trauma[Title/Abstract] OR occupational stress[Title/Abstract] OR secondary traumatic stress[Title/Abstract] OR vicarious trauma[Title/Abstract] OR compassion fatigue[Title/Abstract] OR moral injury[Title/Abstract] OR posttraumatic stress[Title/Abstract] OR post-traumatic stress[Title/Abstract] OR PTSD[Title/Abstract] OR cumulative trauma[Title/Abstract] |
| S6 | S4 OR S5 |
| S7 | "Family"[Mesh] OR "Spouses"[Mesh] OR "Child"[Mesh] |
| S8 | famil*[Title/Abstract] OR family member*[Title/Abstract] OR spouse*[Title/Abstract] OR partner*[Title/Abstract] OR husband*[Title/Abstract] OR wife[Title/Abstract] OR wives[Title/Abstract] OR child[Title/Abstract] OR children[Title/Abstract] OR adolescent*[Title/Abstract] OR parent*[Title/Abstract] OR mother*[Title/Abstract] OR father*[Title/Abstract] OR sibling*[Title/Abstract] OR caregiver*[Title/Abstract] OR household member*[Title/Abstract] OR significant other*[Title/Abstract] |
| S9 | S7 OR S8 |
| S10 | S3 AND S6 AND S9 |
| Limits | English language; database inception to the final search date |

Search notes: The search strategy combines controlled vocabulary (MeSH) and free-text keywords. Boolean operators (AND/OR), truncation (*), phrase searching, and field-specific searching (Title/Abstract) are used to maximize sensitivity and specificity. The search strategy will be adapted for PsycINFO, CINAHL, Web of Science Core Collection, and Scopus using each database's controlled vocabulary and search syntax.

**Adaptation of Search Strategies for Additional Databases**

The PubMed (MEDLINE) search strategy presented above served as the reference strategy for the planned electronic database searches. Equivalent search strategies will be developed for PsycINFO, CINAHL, Web of Science Core Collection, and Scopus by adapting database-specific controlled vocabulary, subject headings, indexing terms, field tags, Boolean operators, truncation, phrase searching, and search syntax while maintaining conceptual equivalence across the three core search concepts. Adaptation of the search strategy will be undertaken in consultation with an academic librarian with expertise in systematic and scoping review methodology to ensure methodological consistency, transparency, reproducibility, and optimal search sensitivity across databases. This approach is consistent with best-practice guidance for the development and reporting of electronic search strategies, including the Peer Review of Electronic Search Strategies (PRESS) Guideline Statement and relevant elements of the PRISMA-S reporting guideline.

Draft search strategies for PsycINFO, CINAHL, Web of Science Core Collection, and Scopus, adapted from the PubMed (MEDLINE) reference strategy, are presented below. These will be finalized in consultation with an academic librarian prior to execution of the comprehensive search.

**PsycINFO (EBSCOhost)**

Platform: EBSCOhost | Coverage: Database inception to the final search date | Language: English

| **No.** | **Search Strategy** |
| --- | --- |
| S1 | DE "Health Personnel" OR DE "Emergency Medical Technicians" OR DE "Firefighters" OR DE "Police Personnel" OR DE "Social Workers" |
| S2 | TI (frontline worker* OR front-line worker* OR first responder* OR emergency responder* OR healthcare worker* OR health care worker* OR nurse* OR paramedic* OR firefighter* OR police officer* OR correctional officer* OR social worker* OR humanitarian worker*) OR AB (same) |
| S3 | S1 OR S2 |
| S4 | DE "Posttraumatic Stress Disorder" OR DE "Occupational Stress" |
| S5 | TI (trauma* OR "traumatic stress" OR "occupational trauma" OR "duty-related trauma" OR "secondary traumatic stress" OR "vicarious trauma" OR "compassion fatigue" OR "moral injury" OR PTSD OR "cumulative trauma") OR AB (same) |
| S6 | S4 OR S5 |
| S7 | DE "Family Members" OR DE "Spouses" OR DE "Children" |
| S8 | TI (famil* OR spouse* OR partner* OR child* OR parent* OR sibling* OR caregiver* OR "household member*") OR AB (same) |
| S9 | S7 OR S8 |
| S10 | S3 AND S6 AND S9 |
| Limits | English language; database inception to the final search date |

**CINAHL (EBSCOhost)**

Platform: EBSCOhost | Coverage: Database inception to the final search date | Language: English

| **No.** | **Search Strategy** |
| --- | --- |
| S1 | (MH "Health Personnel+") OR (MH "Emergency Medical Technicians") OR (MH "Firefighters") OR (MH "Police") OR (MH "Social Workers") |
| S2 | TI (frontline worker* OR first responder* OR healthcare worker* OR nurse* OR paramedic* OR firefighter* OR police officer* OR correctional officer* OR social worker*) OR AB (same) |
| S3 | S1 OR S2 |
| S4 | (MH "Stress Disorders, Post-Traumatic") OR (MH "Occupational Stress") |
| S5 | TI (trauma* OR "traumatic stress" OR "secondary traumatic stress" OR "vicarious trauma" OR "compassion fatigue" OR "moral injury" OR PTSD) OR AB (same) |
| S6 | S4 OR S5 |
| S7 | (MH "Family") OR (MH "Spouses") OR (MH "Child") |
| S8 | TI (famil* OR spouse* OR partner* OR child* OR parent* OR sibling* OR caregiver*) OR AB (same) |
| S9 | S7 OR S8 |
| S10 | S3 AND S6 AND S9 |
| Limits | English language; database inception to the final search date |

**Web of Science Core Collection**

Platform: Clarivate Web of Science | Coverage: Database inception to the final search date | Language: English

TS=(("frontline worker*" OR "front-line worker*" OR "first responder*" OR "healthcare worker*" OR nurse* OR paramedic* OR firefighter* OR "police officer*" OR "correctional officer*" OR "social worker*" OR "humanitarian worker*")) AND TS=((trauma* OR "traumatic stress" OR "occupational trauma" OR "secondary traumatic stress" OR "vicarious trauma" OR "compassion fatigue" OR "moral injury" OR PTSD OR "cumulative trauma")) AND TS=((famil* OR spouse* OR partner* OR child* OR parent* OR sibling* OR caregiver* OR "household member*")) AND LA=(English)

*Limits: Language limited via LA=(English); no date restriction applied (default database inception to final search date).*

**Scopus**

Platform: Elsevier Scopus | Coverage: Database inception to the final search date | Language: English

TITLE-ABS-KEY(("frontline worker*" OR "front-line worker*" OR "first responder*" OR "healthcare worker*" OR nurse* OR paramedic* OR firefighter* OR "police officer*" OR "correctional officer*" OR "social worker*")) AND TITLE-ABS-KEY((trauma* OR "traumatic stress" OR "occupational trauma" OR "secondary traumatic stress" OR "vicarious trauma" OR "compassion fatigue" OR "moral injury" OR PTSD)) AND TITLE-ABS-KEY((famil* OR spouse* OR partner* OR child* OR parent* OR sibling* OR caregiver*)) AND LANGUAGE(english)

*Limits: Language limited via LANGUAGE(english); no date restriction applied (default database inception to final search date).*
