# Supplementary material for: Family Outcomes Associated With Occupational Trauma Exposure in Frontline Workers: Protocol for a Scoping Review
Source: JMIR Res Protoc. 2026 Sep 16;15:e93712. doi: 10.2196/93712 (PMC13581144; doi:10.2196/93712)
Supplement: Supplementary file 2 [file resprot-v15-e93712-s002.docx]

**Checklist 1**

**Preferred Reporting Items for Systematic Reviews and Meta-Analyses Extension for Scoping Reviews (PRISMA-ScR) Checklist**

*Family Outcomes Associated with Occupational Trauma Exposure in Frontline Workers: A Scoping Review Protocol*

| **Section** | **Item** | **PRISMA-ScR Checklist Item** | **Reported (Y/N/N/A)** |
| --- | --- | --- | --- |
| **TITLE** | | | |
| Title | 1 | Identify the report as a scoping review. | Y – Title identifies the report as a “Scoping Review Protocol.” |
| **ABSTRACT** | | | |
| Structured summary | 2 | Provide a structured summary that includes (as applicable): background, objectives, eligibility criteria, sources of evidence, charting methods, results, and conclusions that relate to the review questions and objectives. | Y – Abstract includes Background, Objective, Methods, Results, and Conclusions. |
| **INTRODUCTION** | | | |
| Rationale | 3 | Describe the rationale for the review in the context of what is already known. Explain why the review questions/objectives lend themselves to a scoping review approach. | Y – Introduction. |
| Objectives | 4 | Provide an explicit statement of the questions and objectives being addressed with reference to their key elements (e.g., population or participants, concepts, and context) or other relevant key elements used to conceptualize the review questions and/or objectives. | Y – Scoping Review Questions section. |
| **METHODS** | | | |
| Protocol and registration | 5 | Indicate whether a review protocol exists; state if and where it can be accessed (e.g., a Web address); and if available, provide registration information, including the registration number. | Y – This document is the protocol. Not registered in PROSPERO, as PROSPERO does not routinely accept scoping review protocols and prospective registration is not required for this review type. |
| Eligibility criteria | 6 | Specify characteristics of the sources of evidence used as eligibility criteria (e.g., years considered, language, and publication status), and provide a rationale. | Y – Eligibility Criteria section (Population, Concept, Context, Date of Publication, Language, Types of Evidence Source). |
| Information sources | 7 | Describe all information sources in the search (e.g., databases with dates of coverage and contact with authors to identify additional sources), as well as the date the most recent search was executed. | Y – Search Strategy section names all five databases and coverage (inception to final search date); exact final search date to be confirmed upon execution of comprehensive searches. |
| Search | 8 | Present the full electronic search strategy for at least 1 database, including any limits used, such that it could be repeated. | Y – Full PubMed (MEDLINE) strategy provided in Multimedia Appendix 2 (Search Strategy). |
| Selection of sources of evidence | 9 | State the process for selecting sources of evidence (i.e., screening and eligibility) included in the scoping review. | Y – Sources of Evidence section describes two-stage screening process. |
| Data charting process | 10 | Describe the methods of charting data from the included sources of evidence (e.g., calibrated forms or forms that have been tested by the team before their use, and whether data charting was done independently or in duplicate) and any processes for obtaining and confirming data from investigators. | Y – Data Extraction section. |
| Data items | 11 | List and define all variables for which data were sought and any assumptions and simplifications made. | Y – Table 1 (Planned Data Charting Framework). |
| Critical appraisal of individual sources of evidence | 12 | If done, provide a rationale for conducting a critical appraisal of included sources of evidence; describe the methods used and how this information was used in any data synthesis (if appropriate). | N/A – Not undertaken, consistent with JBI guidance and PRISMA-ScR for scoping reviews; rationale stated in Data Extraction section. |
| Synthesis of results | 13 | Describe the methods of handling and summarizing the data that were charted. | Y – Data Synthesis section. |
| **RESULTS** | | | |
| Selection of sources of evidence | 14 | Give numbers of sources of evidence screened, assessed for eligibility, and included in the review, with reasons for exclusions at each stage, ideally using a flow diagram. | N/A – This is a protocol; comprehensive database searches, screening and study selection have not yet been undertaken and will follow publication of this protocol. |
| Characteristics of sources of evidence | 15 | For each source of evidence, present characteristics for which data were charted and provide the citations. | N/A – Not yet available; searches not yet conducted. |
| Critical appraisal within sources of evidence | 16 | If done, present data on critical appraisal of included sources of evidence (see item 12). | N/A – Critical appraisal not undertaken (see item 12). |
| Results of individual sources of evidence | 17 | For each included source of evidence, present the relevant data that were charted that relate to the review questions and objectives. | N/A – Not yet available; searches not yet conducted. |
| Synthesis of results | 18 | Summarize and/or present the charting results as they relate to the review questions and objectives. | N/A – Not yet available; searches not yet conducted. |
| **DISCUSSION** | | | |
| Summary of evidence | 19 | Summarize the main results (including an overview of concepts, themes, and types of evidence available), link to the review questions and objectives, and consider the relevance to key groups. | Y – Discussion: Principal Findings (anticipated significance, based on preliminary scoping searches). |
| Limitations | 20 | Discuss the limitations of the scoping review process. | Y – Discussion: Strengths and Limitations. |
| Conclusions | 21 | Provide a general interpretation of the results with respect to the review questions and objectives, as well as potential implications and/or next steps. | Y – Conclusions section and Discussion: Future Directions. |
| **FUNDING** | | | |
| Funding | 22 | Describe sources of funding for the included sources of evidence, as well as sources of funding for the scoping review. Describe the role of the funders of the scoping review. | Y – Funding section: this study did not receive any specific funding. |

JBI = Joanna Briggs Institute; PRISMA-ScR = Preferred Reporting Items for Systematic Reviews and Meta-Analyses Extension for Scoping Reviews.

Items 14–18 (Results) are marked N/A because this document is a protocol; comprehensive database searches, screening, data charting, and synthesis had not yet been undertaken at the time of writing and will follow publication of this protocol.
